# An Adaptive Bandwidth Management Algorithm for Next-Generation Vehicular Networks

**DOI:** 10.3390/s23187767

**Published:** 2023-09-08

**Authors:** Chenn-Jung Huang, Kai-Wen Hu, Hao-Wen Cheng

**Affiliations:** 1 Department of Computer Science & Information Engineering, National Dong Hwa University, Shoufeng, Hualien County 974301, Taiwan; 2586611811v@gmail.com; 2 Lookout, Inc., Taipei 110207, Taiwan; drive55555@gmail.com

**Keywords:** electric vehicle, bandwidth allocation, video service, 6G, data mining, machine learning, optimization

## Abstract

The popularity of video services such as video call or video on-demand has made it impossible for people to live without them in their daily lives. It can be anticipated that the explosive growth of vehicular communication owing to the widespread use of in-vehicle video infotainment applications in the future will result in increasing fragmentation and congestion of the wireless transmission spectrum. Accordingly, effective bandwidth management algorithms are demanded to achieve efficient communication and stable scalability in next-generation vehicular networks. To the best of our current knowledge, a noticeable gap remains in the existing literature regarding the application of the latest advancements in network communication technologies. Specifically, this gap is evident in the lack of exploration regarding how cutting-edge technologies can be effectively employed to optimize bandwidth allocation, especially in the realm of video service applications within the forthcoming vehicular networks. In light of this void, this paper presents a seamless integration of cutting-edge 6G communication technologies, such as terahertz (THz) and visible light communication (VLC), with the existing 5G millimeter-wave and sub-6 GHz base stations. This integration facilitates the creation of a network environment characterized by high transmission rates and extensive coverage. Our primary aim is to ensure the uninterrupted playback of real-time video applications for vehicle users. These video applications encompass video conferencing, live video, and on-demand video services. The outcomes of our simulations convincingly indicate that the proposed strategy adeptly addresses the challenge of bandwidth competition among vehicle users. Moreover, it notably boosts the efficient utilization of bandwidth from less crowded base stations, optimizes the fulfillment of bandwidth prerequisites for various video applications, and elevates the overall video quality experienced by users. Consequently, our findings serve as a successful validation of the practicality and effectiveness of the proposed methodology.

## 1. Introduction

As the greenhouse effect worsens, environmental regulations for vehicles are now becoming more stringent in numerous countries. In order to meet the increasingly stringent regulations on greenhouse gas emissions and to solve the problems associated with traditional engine vehicles, such as air pollution, climate change, and high fuel prices, electric vehicles (EV), which use green energy to reduce carbon emissions, have been recognized as the future alternative to traditional automobiles.

Affected by the COVID-19 epidemic that started in late 2019, academics and organizations have been turning to video conferencing to match the COVID-19 precautionary measures, as well as to improve efficiency and convenience. Video conferencing applications account for an increasing share of Internet applications and bandwidth usage [1]. Nowadays, video conferencing plays an important role in people’s lives. As video conferencing requires real-time transmission, the issue of resource allocation of uplinks is of concern. If a large amount of data is uploaded at the same time during rush hours, the smoothness and stability of the video conferencing may be affected. In addition, multimedia streaming plays a key role in various emerging in-vehicle infotainment applications [2]. The rise of video service providers such as TikTok and YouTube has emphasized the popularity of video services in everyday life. Video traffic has been growing in recent years [3], and these video service providers offer video on demand (VoD) streaming services to users through websites, mobile apps, or social networks [4]. Live video has also become one of the most popular consumer contents, such as online education, trade shows, sports events, concerts, and video games [5]. With the advancement of self-driving technology, autonomous electric vehicles are bound to become the mainstream of human transportation in the future, which will enable people to spend their time in the vehicle on entertainment and office work.

Encoding plays a vital role in modern digital media, ensuring efficient and reliable transmission and playback of videos in various situations. Currently, a significant portion of videos use Advanced Video Coding (AVC) [6] as their chosen encoding method, which was introduced in 2003 and is one of the most commonly used video coding standards. It is widely applied in various applications, such as video conferencing, mobile services, and high-definition video storage [7]. While a subsequent development, namely, High-Efficiency Video Coding (HEVC) [8], did lead to a notable reduction in the bitrate for 4K videos, it still proved insufficient in terms of efficiency for 8K applications [9]. The latest international video coding standard, Versatile Video Coding (VVC) [10], was introduced in 2020. Compared to HEVC, VVC offers higher compression efficiency [11]. VVC adopts various new coding technologies [12]. Multiple coding unit partitions and numerous coding tools improve compression performance, but also greatly increase coding complexity [13]. There are already studies comparing the performance of different codecs. Menasri and Skoudarli [14] proposed a performance comparison of throughput between context-based adaptive binary arithmetic decoding processes adopted in the AVC, HEVC, and VVC. Bonnineau et al. [9] evaluated 8K videos using HEVC and VVC, noting that VVC resulted in an average bitrate reduction of around 41%. Choi [15] conducted a comparison of the complexity between HEVC and VVC, revealing that VVC’s encoding time was up to 27 times greater than HEVC in certain instances. Belda et al. [16] compared the encoding time of VVC, AVC, and HEVC. Their experimental results demonstrated that the encoding delay of VVC was significantly higher than that of AVC and HEVC. Accordingly, the above-mentioned experimental results indicate that VVC is not suitable for real-time transmission application demands.

Commencing in 2019, the official deployment of 5G witnessed the incorporation of sub-6 GHz and millimeter wave (mmWave) frequencies [17]. In response to the dramatic increase in video traffic, several technologies were employed to alleviate traffic burdens. However, if all video requests are served through wired backhaul links during rush hours, it would burden the wireless transmission channels heavily, leading to capacity bottlenecks for wireless video traffic [18]. Moreover, traditional data transmission within networks faces several limitations, including elevated latency, significant packet loss, and network congestion. These challenges must be effectively addressed by the next generation of wireless networks. Liu et al. [19] introduced a dual-layer algorithm to tackle the transmission challenge within a mobile wireless-powered communication network. The algorithm’s primary objective is to optimize throughput by strategically pairing the energy consumption of a single transmission with the energy harvesting probability. Zheng et al. [20] conducted a study on the radio frequency-powered ambient backscatter-assisted hybrid underlay cognitive radio network. An adjusted deep deterministic policy gradient algorithm was proposed to establish an effective policy for time scheduling and energy management, with the ultimate aim of optimizing long-term secondary throughput. Furthermore, they integrated convex optimization into this algorithm to accelerate convergence and identify the optimal solution. Mei et al. [21] successfully adapted to the dynamic nature of the system and upheld stability in connection to task queues and battery levels through the application of Lyapunov optimization theory. They formulated an algorithm with the primary objective of optimizing system throughput to its maximum capacity. Wang et al. [22] placed their emphasis on effective throughput as the primary performance metric. They not only derived the mathematical expression for effective throughput, but also posed an optimization challenge with the objective of maximizing it.

Beyond tackling the issues of the next-generation network by enhancing network architecture and optimizing algorithms for resource allocation, the forthcoming generation of networks will predominantly revolve around 6G technology. Thus, 6G promises to usher in an era characterized by continuous connectivity, immersive experiences, robust support for a multitude of simultaneous users, ultra-low latency, universal accessibility, unmatched data capacity, unwavering reliability, and stringent security measures [23]. In recent literature, Pei et al. [24] introduced several pivotal technologies aimed at facilitating the implementation of 6G. They anticipated that 6G networks would not only rely on conventional spectrum usage, but also explore previously uncharted frequency bands within the context of cellular communication standards. These unexplored bands specifically included the terahertz (THz) frequency band and visible light communication (VLC). Expanding on these emerging concepts, the investigation of the THz frequency band has garnered significant attention in recent years. This untapped realm holds the potential to reshape the landscapes of wireless communication. The THz band occupies a frequency range situated between microwave and infrared within the electromagnetic spectrum. Its frequencies span from 300 GHz to 3 THz, accompanied by wavelengths ranging from 1 mm to 0.1 mm [25]. In order to support potential applications related to future vehicles, vehicle communication demands higher reliability and lower latency for transmitting large amounts of data. The THz band can provide ultra-high-speed data transmission, large bandwidth, and extremely low latency [26].

Scholars have already conducted research on the applications of THz in vehicle communications. Li et al. [27] analyzed vehicular communications in a 300 GHz urban scenario, providing a detailed characterization of path loss, shadow fading, and other properties. Lin et al. [28] addressed vehicle tracking and resource allocation for THz vehicle-to-infrastructure communication networks, proposing a solution using the Unscented Kalman Filter. Moltchanov et al. [29] established a mathematical framework for comparing multi-hop relaying systems with antennas installed at different positions on vehicles using IEEE 802.15.3d parameters and 300 GHz propagation measurements. They claimed that placing the antenna on the windshield effectively reduces the sensitivity of the technology penetration rate and increases transmission coverage. However, THz suffers from severe attenuation caused by molecular absorption due to its wavelength size being similar to atmospheric particles like raindrops and dust [30]. Additionally, THz waves encounter spreading loss caused by electromagnetic wave diffusion in the medium. As the transmission distance increases, the path loss from absorption and diffusion becomes more significant [31], limiting the long-range transmission capability of THz.

To address these limitations, the dense deployment of base stations is required to extend coverage. Moreover, the expenses associated with THz equipment are substantial [32]. To extensively implement THz base stations, telecommunication providers would be required to make substantial financial investments. Researchers have proposed deploying base stations in different frequency bands to overcome the transmission challenges of high-frequency base stations. Kouzayha et al. [33] deployed THz base stations for high data rates and radio frequency base stations for coverage. Wang and Chun [34] introduced a hybrid network architecture that includes both THz and mmWave frequencies. Moltchanov et al. [35] reviewed communication network deployment, propagation, antennas, blockage, micro mobility, beam searching, traffic, and service models. They explored the deployment and transmission challenges of mmWave and THz base stations in urban environments, considering different system and environmental conditions along with base stations operating at THz, mmWave, and sub-6 GHz frequencies. Yin et al. [36] deployed mmWave base stations to overcome the limited bandwidth of traditional microwave frequencies. They densely deployed mmWave base stations in existing base stations to form a heterogeneous mmWave network.

Alongside the THz frequency range, another set of frequencies currently unutilized in existing communication standards is VLC. This alternative spectrum brings forth specific advantages over other communication technologies. Firstly, VLC is relatively safe for human eyes [37] and can be easily integrated with existing LED lighting infrastructure, such as streetlights, traffic lights, and vehicle headlights, making it a convenient communication transmitter [38]. VLC not only integrates communication and lighting, but also presents benefits such as low power consumption, the utilization of a license-free spectrum, strong security, and resilience to electromagnetic interference [39]. With its limited field of view and line-of-sight transmission, VLC is well-suited for high-speed short-range wireless communication [40]. This makes VLC particularly suitable for vehicle-to-vehicle (V2V) communication, especially for applications requiring highly secure, reliable, and low-latency communication between vehicles, such as vehicle platoons [41]. Recently, Aghaei et al. [42] undertook a comprehensive analysis that contrasted the distinctions and benefits of VLC and mmWave within V2V communication. They presented the received signal strengths of both systems, delved into channel characteristics for inter-vehicle communication, and evaluated the impact of vehicle density on communication efficacy. Kamiya et al. [43] showcased the effective capturing of VLC signals during vehicular movement at a velocity of 40 km/h. The transmission occurred via an LED array, with the signals being received by an image sensor employing a rolling shutter mechanism. This investigation affirmatively established the feasibility of receiving VLC signals while in motion. Yang et al. [44] delineated the challenges linked with vehicular VLC and proposed an inventive architecture featuring tracking and environment sensing capabilities. The results of their simulations provided validation for the effectiveness of the proposed VLC system, showcasing its potential to achieve a bit error rate below $10^{-4}$, even when confronted with substantial interference from external lighting sources. Additionally, a multiple input and multiple output (MIMO) VLC system with custom-designed pin arrays and headlights achieved data rates of 336 Mbps and 362 Mbps at distances of 100 m during the day and night, respectively [45]. The above-mentioned studies verify the feasibility of VLC in next-generation vehicular communication applications.

Beyond the realm of 6G technology, the significance of edge computing has risen substantially within contemporary communication networks. This strategy, involving the proximity of computation and resources to users, harmonizes seamlessly with the high-speed and low-latency benefits inherent in 6G. It is foreseeable that within the landscape of vehicular networks in the years to come, the fusion of 6G technology and edge computing will engender heightened efficacy in data processing and real-time applications. The advancement of mobile edge computing has also inspired the development of edge transcoding technology, where service providers can distribute transcoding tasks from central servers to edge servers closer to users for video transcoding. Edge transcoders can convert high-bitrate video into low-bitrate versions for user selection, improving the performance of video services by reducing transmission delay [46]. Furthermore, lightweight edge computing servers can be deployed at edge nodes like base stations. Video files can be stored on these servers for users to download, and if THz base stations are used, video transmission can become more efficient and faster, benefiting the provision of high-quality video services and meeting user demands [47]. Moreover, user equipment’s computing and storage capabilities have significantly improved, enabling their participation in edge computing, storage, and communication as well [48].

As the demand for future multimedia traffic continues to rise, bandwidth shortages become unavoidable during peak periods. At present, multiple research endeavors have introduced techniques to allocate bandwidth and improve mobile network throughput, aiming to tackle the challenge of allocating network resources efficiently among users. Yuan et al. [49] introduced an algorithm supporting mobile video streaming applications in heterogeneous wireless networks. Their research allocated bandwidth among multiple users based on user experience quality and mobility-related information, enhancing user experience quality through a push strategy using the HTTP/3 protocol. Liu et al. [50] designed a federated deep reinforcement learning-based video streaming scheduling algorithm. Their algorithm predicted reasonable bandwidth allocation weights based on the current player’s state and information provided by servers, subsequently allocating available bandwidth. Tung and Gündüz [51] utilized deep neural networks for end-to-end compression and channel coding of video frames, optimizing video frame bandwidth allocation through reinforcement learning.

The advent of 6G technology holds the promise of significantly enhancing vehicular mobility. Vehicles operating at high speeds can leverage the capabilities of 6G connectivity to establish cooperative communication, thereby ensuring safe following distances and smooth traffic flow [52]. Consequently, as we enter the era of 6G networks, the optimization of bandwidth allocation among vehicles is poised to become a pivotal concern. In recent years, a plethora of studies have surfaced, introducing inventive bandwidth allocation schemes customized for multimedia applications within vehicular networks. Xiong et al. [53] proposed an algorithm based on predictions of the vehicular communication and edge computing network states. It optimizes communication resource allocation, transmission paths, and power consumption for computation modes. Zhang et al. [54] employed radar communication rates, bandwidth allocation, and base station selection as parameters, utilizing reinforcement learning techniques to handle uncertainty in vehicle movement and fluctuations in multimedia data volume. Yun et al. [55] presented a video streaming strategy tailored for mobility-aware vehicular networks, harnessing the power of deep reinforcement learning. Within this scheme, millimeter-wave base stations were harnessed for the delivery of videos to users. Additionally, it crafted a dynamic video delivery approach that intelligently determined the content, quality, and quantity of video chunks. Cheng et al. [56] devised a cost-efficient task processing scheme for a dual-band cooperative vehicular network. This scheme allowed tasks to be processed locally or offloaded to either the macro-cell base station or road-side units (RSU). By optimizing task scheduling, computation, and communication resource allocation, while taking into account the vehicle’s sojourn time, they aimed to minimize the total cost, considering both energy consumption and latency. Jiang et al. [57] conducted a comprehensive review of resource allocation strategies used for video streaming in vehicular ad hoc networks. They also explained in detail the widely adopted and practical optimization tools. Furthermore, they summarized the technologies that enable video streaming over vehicular ad hoc networks, with a particular focus on the integration of video communication, caching, and computing. Dai et al. [2] introduced a mobile edge computing-based framework for adaptive-bitrate multimedia streaming within the internet of vehicles. They employed deep Q-learning (DQN) to enhance solutions by revisiting past experiences and updating Q-functions using gradients. They also presented an adaptive-quality-based chunk selection (AQCS) algorithm that factored in service quality, available playback time, and freezing delay to determine both bandwidth allocation and video quality levels.

Based on the literature cited above, it is evident that most recent studies have primarily focused on RSUs and base stations for video downloading and transmission, while overlooking the potential of vehicular communication technology to enhance these processes and neglecting the prioritization of real-time applications. With the expanding user base of video applications, it is apparent that perceived video quality could be adversely affected by bandwidth constraints during peak traffic hours. In view of different characteristics and requirements of multimedia applications, future bandwidth allocation schemes should be planned according to the multimedia requirements so that the real-time multimedia applications can be prioritized to receive sufficient bandwidth.

To the best of our knowledge, there is a noticeable gap in the existing literature when it comes to exploring the utilization of the most recent 6G network communication technologies for distributing bandwidth in multimedia applications within upcoming vehicular networks. In light of this, our paper employs THz base stations, as well as mmWave and sub-6 GHz band base stations, to fulfill the bandwidth requirements for video applications targeting EV users. Due to the continuous growth of video traffic, service providers need to offer high-quality streaming approaches to meet user expectations. In addition, this paper adopts EVs as edge devices, enabling them to provide edge computing, storage, and communication capabilities. VLC technology is being utilized in V2V communications to provide high data rate and high security for inter-vehicle video transmissions. Considering the different characteristics and requirements of multimedia applications, this paper employs the most widely used AVC with high encoding speed for real-time video conferencing sessions and live videos, and adopts the VVC with high compression rate for non-real-time VoDs, so that the users can watch high-resolution videos at a lower bit rate.

In view of the significant time delay of traditional centralized computing for multimedia applications during peak hours and the increasing computational power of edge nodes, a decentralized computing architecture is adopted in this paper.

The main contributions of this paper can be outlined as follows:

- Through the utilization of the fundamental technologies of 6G, such as THz frequencies, in conjunction with the existing 5G mmWave and sub-6 GHz base stations, this paper facilitates the creation of a network environment characterized by elevated transmission rates and extensive coverage.
- In order to ensure uninterrupted real-time video playback for EV users, this paper chooses the low-latency AVC method as the preferred approach. This decision is especially crucial for applications such as real-time video conferencing and live video. Moreover, the introduced algorithm places high priority on efficiently allocating bandwidth for real-time video content. All of these efforts are directed towards achieving the best possible performance standards for users of EVs.
- This paper demonstrates the capability to employ VLC for V2V communication, thereby enabling the redistribution of bandwidth from less-congested base stations located in alternate road sections. Furthermore, this paper capitalizes on base stations equipped with ample bandwidth and fosters collaboration among other EVs to pre-download video segments. This strategic approach leads to noteworthy improvements in bandwidth availability for various video applications, while also markedly enhancing the efficient utilization of bandwidth resources from less-congested base stations. Additionally, this paper successfully mitigates prolonged delays in downloading video content from distant servers and alleviates congestion in vehicular networks arising from a substantial influx of video applications by EV users during peak hours. Consequently, this paper effectively heightens users’ perceived video quality across all genres of video applications.

Section 2 provides an extensive outline of the architecture underlying our envisioned next-generation vehicular networks. It also furnishes an elaborate elucidation of the techniques and processes undertaken by every module within the proposed algorithm. Moving on, Section 3 evaluates the effectiveness of the newly introduced algorithm. Lastly, Section 4 concludes the study and engages in a thorough discussion of the findings.

## 2. Research Methodology and Steps of the Study

**Figure 1:**
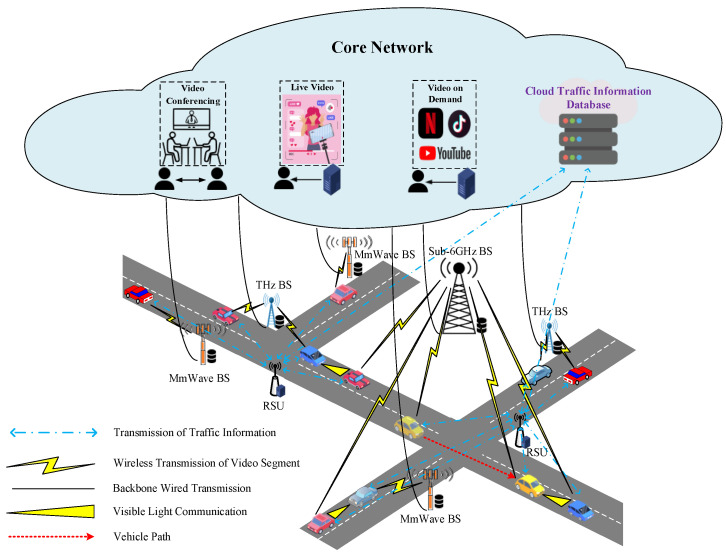
Sample scenario of video transmissions for next-generation vehicular networks.

This paper employs a decentralized computing architecture, as illustrated in Figure 1. This approach aims to mitigate the computational complexity inherent in the conventional centralized control framework. Each EV sets up its route before starting the journey. The RSU managing a road section is responsible for the allocation of video bandwidth for each passing EV according to the video application requirements of each EV user. If an EV user’s video is unable to meet the bandwidth requirements for certain congested road sections, the EV will request the RSU managing the road section to assist in scheduling the required bandwidth for the video application. The foundation of this paper rests on the categorization of video applications into three distinct types: video conferencing, live video, and VoD. As mentioned above, video conferencing and live video use the current mainstream AVC [6], while VoD uses VVC [10] with half the bit rate of the HEVC, and can provide high resolution video, such as 4K/8K video, if needed. A VoD can be pre-converted into multiple bit rate versions. Before each video segment is played, the video segment that meets the bit rate requirement is downloaded to the on-board storage of the EV according to the preference set by the video user.

**Figure 2:**
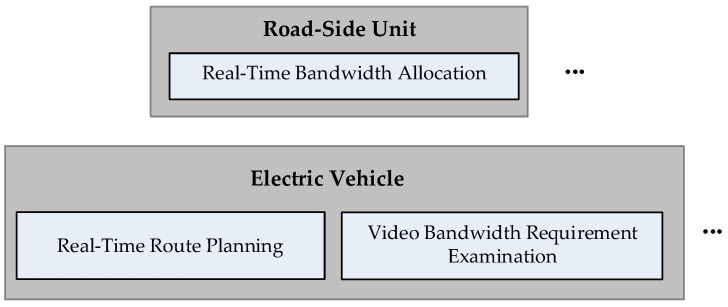
Schematic diagram of the proposed algorithm.

Figure 2 shows the video bandwidth allocation architecture for the video conferencing, live video and VoD applications used by EV passengers. The “Real-time Route Planning” module installed within the on-board unit (OBU) of each EV is activated to set the route before the EV departs, and then the route is transmitted to the RSU that manages the road sections along the way. When an EV user activates a video application while the vehicle is in motion, the “Video Bandwidth Requirement Examination” module, configured within the OBU, establishes a connection with the video application software provider’s server to obtain the video specification information according to the pre-set video quality requirements of the EV user, and confirms whether sufficient bandwidth can be obtained from the base station coverage area of the roadway along which the EV travels. In this paper, the aforementioned three types of base stations, namely, THz, mmWave, and sub-6 GHz, are used to provide bandwidth for video applications on the roadways where EVs travel.

In the realm of advancing chip technology, there is a prevailing anticipation that forthcoming next-generation vehicular networks will prominently integrate chips for a diverse range of computational functions. Addressing the burgeoning demand for decentralized computing, as introduced in this paper, dedicated servers are strategically deployed at both RSUs and base stations. Their primary purpose is to facilitate multimedia processing and computational transmissions.

Within the context of RSUs, these servers assume a pivotal role in several key aspects. They diligently record the bandwidth requirements of electric vehicle users within their respective managed segments. Concurrently, these servers store crucial information regarding the available bandwidth originating from base stations. Moreover, these servers harness their computational prowess to efficiently execute various RSU modules, optimizing their operational performance.

On the flip side, base stations also reap the benefits of these servers. Once an EV user initiates a video service application, the server situated at the base station undertakes the task of intelligently prioritizing the storage of specific video files. This strategic maneuver effectively mitigates potential download latency concerns, enhancing the overall user experience. Additionally, leveraging the robust computational capabilities of these servers, video transcoding emerges as a feasible capability. This translates to offering users an array of video resolutions to choose from, tailored to their preferences and requirements.

If the coverage area of the base station of the road section that the EV traverses cannot meet the bandwidth requirement for the EV user’s video application, the managing RSU of the road section will activate the “Real-time Bandwidth Allocation” module to assist in allocating the bandwidth to meet the minimum requirement for the video application according to the characteristics of the video application and the pre-set video quality requirement of the EV user. In the case of video conferencing or live video, the RSU starts with the EV, adds other EVs arriving at the road section at the same time to form a fleet, and continues to expand the fleet until the last fleet member is on a road section where the base station’s coverage can provide bandwidth to the requesting EV. For the scenario involving VoD, this paper proposes a strategy wherein a separate EV traversing the same road segment pre-downloads the necessary video segment when in a bandwidth-accessible area. Subsequently, when the two EVs cross paths, the pre-downloaded video segment is transmitted from the previously prepared EV to the requesting EV user via V2V communication. Notably, the EV using the video application will also track the EV users’ satisfaction with the video playback quality at regular intervals during the video playback process, and if there is a need to adjust the video playback quality, it will notify the “Real-time Bandwidth Allocation” module of the managing RSU(s) for subsequent bandwidth adjustments of the video usage.

The modules shown in Figure 2 are described below.

### 2.1. Real-Time Route Planning for EVs

Before the start of the EV, the EV user starts the OBU to set the departure point, departure time, and destination, and then starts to run this module. The EV first downloads the global real-time road traffic information from the cloud, and uses Dijkstra’s algorithm to estimate the shortest route from the departure point to the destination based on the average traveling time of each road section. Since the arrival time of EVs at each road segment is affected by the traffic conditions at the time of arrival at the road section, this module calculates the arrival time at each road section based on the latest traffic condition information of each road segment of the shortest driving route, and notifies the RSU managing the road section of the driving route and the estimated time of arrival at each road section.

To avoid discrepancies between the latest estimated arrival times of EVs and their expected arrival times at road sections due to ad hoc changes in itineraries by EV users or road congestion during peak hours, this module recalculates the arrival time at each road section at regular intervals based on the latest traffic condition information sent from the RSU that manages each road section the EV travels through, and uses a machine learning technique to recalculate the arrival time at each road section. If the recalculated arrival times at the road sections are too different from the original predicted times, the module sends the updated times to the RSUs that manage the road sections of the traveled route. Given the effectiveness of machine learning in predicting road travel times in the literature [58,59], the support vector regression (SVR) technique [60] is used in this paper to predict the arrival time at each road section of a traveled route based on the relevant information.

The steps of this module are described below.

Step 1: Before the EV starts its journey, it first downloads the global real-time traffic condition information from the cloud, and after setting the starting location and time as well as the destination, it estimates the shortest path from the starting location to the destination using Dijkstra’s algorithm based on the cost of the average traveling time of each road section.

Step 2: The estimated time of arrival at each road section of the traveled route is given by:

(1) $${at}_{p_{i+1}^{\sigma }}={at}_{p_{i}^{\sigma }}+SVR\left({sp}_{p_{i}^{\sigma },p_{i+1}^{\sigma }}\left({at}_{p_{i}^{\sigma }}\right){,\rho }_{p_{i}^{\sigma },p_{i+1}^{\sigma }}\left({at}_{p_{i}^{\sigma }}\right),{wd}_{p_{i}^{\rho },p_{i+1}^{\rho }}\left({at}_{p_{i}^{\sigma }}\right),{wt}_{p_{i}^{\rho },p_{i+1}^{\rho }}\left({at}_{p_{i}^{\sigma }}\right)\right),1\;\leq \;i\;<\;h^{\sigma },$$

where SVR(·) is the SVR library.

Step 3: The estimated time of arrival at each intersection is transmitted to the RSU that manages each road section, along with the EV’s route.

Step 4: This module performs the background execution mode here. After the preset time interval, the latest traffic condition information is obtained from the RSU managing the road section, and the arrival time at each road section of the traveled route is recalculated using Equation (1).

Step 5: If the updated EV arrives at the traveled section in a time that exceeds the system’s predefined thresholds due to a temporary trip change or peak hour congestion, the RSU governing the traveled section will be notified of the revised arrival time.

Step 6: Before the EV reaches its destination, go back to step 4 to continue.

### 2.2. Video Bandwidth Requirement Examination for EV Users

After an EV user activates the video application while the EV is in motion, this module obtains the requirements and specifications of the application from the server of the video application software provider, and adjusts the quality and bandwidth requirements of the video application in a timely manner according to the user video quality requirements pre-set by the EV user. Then, this module transmits the video bandwidth demand to the managing RSU of each road section that the application travels through during the video usage period, and each RSU carries out the bandwidth allocation according to the demand specification of the video application while the EV traverses on the managing road section.

The steps of this module are described below.

Step 1: After an EV user activates a video application, the requirements and specifications for the video application is retrieved from the video application software vendor’s server.

Step 2: The RSU along the route informs the minimum bandwidth that can be provided by the base station coverage of the road section it manages.

Step 3: Using the driving durations of EVs across individual road sections and considering the resolution prerequisites for video applications by EV users, the calculation of the essential minimum bandwidth required for video application within each time slot or video segment throughout the driving duration takes place. This calculation is followed by an examination of the bandwidth provisioned by the base station within the coverage area of the corresponding road section to ascertain if it aligns with the stipulated requirements of the video application.

(2) $${dub}_{t}^{\sigma ,v}=\sum_{\vartheta }{ub}_{t}^{\vartheta ,\sigma }-\underline{UR}\left({uvr}_{t}^{\sigma ,v}\right),\;\;\;\;\;{at}_{p_{i}^{\sigma }}\;\leq \;t\;<\;{at}_{p_{i+1}^{\sigma }},\;\;1\;\leq \;i\;<\;h^{\sigma },\;if\;\kappa_{1}^{\sigma ,v}=1,$$

(3) $${ddb}_{t}^{\sigma ,v}=\sum_{\vartheta }{db}_{t}^{\vartheta ,\sigma }-\underline{DR}\left({dvr}_{t}^{\sigma ,v}\right),\;{at}_{p_{i}^{\sigma }}\;\leq \;t\;<\;{at}_{p_{i+1}^{\sigma }},\;\;1\;\leq \;i\;<\;h^{\sigma },\;if\;\kappa_{1}^{\sigma ,v}+\kappa_{2}^{\sigma ,v}=1,$$

(4) $${at}_{p_{1}^{\sigma }}\;\leq \;t_{0}^{\sigma ,v}\;\leq \;t_{e}^{\sigma ,v}\;\leq \;{at}_{p_{h^{\sigma }}^{\sigma }},\;if\;\kappa_{1}^{\sigma ,v}+\kappa_{2}^{\sigma ,v}=1,$$

(5) $${at}_{p_{1}^{\sigma }}\;\leq \;{pt}_{1}^{\sigma ,v}\;\leq \;{pt}_{S^{\sigma ,v}}^{\sigma ,v},\;if\;\kappa_{3}^{\sigma ,v}\;=\;1,$$

(6) $${pt}_{s}^{\sigma ,v}\geq {pt}_{s-1}^{\sigma ,v}+{SS}_{s}^{\sigma ,v}\left({br}_{s}^{\sigma ,v}\right)/\sum_{\vartheta }{db}_{\tau_{s}}^{\vartheta ,\sigma },\;{pt}_{1}^{\sigma ,v}\;\leq \;\tau_{s}\;<\;{pt}_{s}^{\sigma ,v},\;1\;<\;s\;\leq \;S^{\sigma ,v},\;if\;\kappa_{3}^{\sigma ,v}=1,$$

(7) $${buf}_{\tau_{s}}^{\sigma }+{SS}_{s}^{\sigma ,v}\left({br}_{s}^{\sigma ,v}\right)\;\leq \;{\bar{buf}}^{\sigma },\;\;{pt}_{1}^{\sigma ,v}\;\leq \;\tau_{s}\;<\;{pt}_{s}^{\sigma ,v},\;\;\;\;1\;<\;s\;\leq \;S^{\sigma ,v},if\;\kappa_{3}^{\sigma ,v}=1.$$

As shown in Equations (2) and (3), if the quantities of ${dub}_{t}^{\sigma ,v}$ and ${ddb}_{t}^{\sigma ,v}$ are greater than zero, it signifies that the base station’s allocated bandwidth within the coverage zone of the route taken by the EV is sufficient to fulfill the requisite minimum upload and download bandwidth criteria for applications such as video conferencing or live video streaming, respectively. Equations (4) and (5) guarantee that the video conferencing or live video initiation time must be scheduled after the EV’s departure time, and the application should be concluded prior to the EV reaching its destination.

Equation (6) clarifies that the pre-download of a video segment must be completed before the playback can begin. Meanwhile, Equation (7) guarantees that the buffer space available in the EV is sufficient to accommodate a video segment prior to its playback.

Step 4: If all of the base station coverage areas of the EV routes can meet the minimum bandwidth requirement, this module notifies each RSU of the road section of the bandwidth used in the road section and proceed to Step 6. On the other hand, this module notifies the RSUs along the road sections with insufficient bandwidth to assist in adjusting the bandwidth. The optimization objectives concerning the video quality requirements of EV users can be formulated by:

(8) $$Max\left\{\kappa_{1}^{\sigma ,v}\cdot \left[\sum_{t_{0}^{\sigma ,v}\;\leq \;t\;\leq \;t_{e}^{\sigma ,v}}\left(QL\left({ur}_{t}^{\sigma ,v}\right)-\phi^{\sigma ,v}\cdot {ult}_{t}^{\sigma ,v}\right)-\xi^{\sigma ,v}\;\;\;\;\;\;\;\;\;\;\;\;\cdot \sum_{t_{0}^{\sigma ,v}\;<\;t\;\leq \;t_{e}^{\sigma ,v}}\left|QL\left({ur}_{t}^{\sigma ,v}\right)-QL\left({ur}_{t-1}^{\sigma ,v}\right)\right|\right]+\left(\kappa_{1}^{\sigma ,v}+\kappa_{2}^{\sigma ,v}\right)\;\;\;\;\;\;\;\;\;\;\;\;\cdot \left[\sum_{t_{0}^{\sigma ,v}\;\leq \;t\;\leq \;t_{e}^{\sigma ,v}}\left(QL\left({dr}_{t}^{\sigma ,v}\right)-\phi^{\sigma ,v}\cdot {dlt}_{t}^{\sigma ,v}\right)-\xi^{\sigma ,v}\;\;\;\;\;\;\;\;\;\cdot \sum_{t_{0}^{\sigma ,v}\;<\;t\;\leq \;t_{e}^{\sigma ,v}}\left|QL\left({dr}_{t}^{\sigma ,v}\right)-QL\left({dr}_{t-1}^{\sigma ,v}\right)\right|\right]+\kappa_{3}^{\sigma ,v}\;\;\;\;\;\;\;\;\;\cdot \left[\sum_{1\;\leq \;s\;\leq \;S^{\sigma ,v}}\left(QD\left({br}_{s}^{\sigma ,v}\right)-\psi^{\sigma ,v}\cdot {rbt}_{s}^{\sigma ,v}\right)-\beta^{\sigma ,v}\;\;\;\;\;\;\;\;\;\;\;\;\;\;\cdot \sum_{1\;<\;s\;\leq \;S^{\sigma ,v}}\left|QD\left({br}_{s}^{\sigma ,v}\right)-QD\left({br}_{s-1}^{\sigma ,v}\right)\right|-\omega^{\sigma ,v}\cdot {sd}^{\sigma ,v}\right]\right\},$$

subject to:

(9) $${ult}_{t}^{\sigma ,v}=\frac{{DS}_{t}^{\sigma ,v}\left(u{vr}_{t}^{\sigma ,v}\right)}{{ur}_{t}^{\sigma ,v}}\;\leq \;{ud}^{\sigma ,v},\;\;t_{0}^{\sigma ,v}\;\leq \;t\;<\;t_{e}^{\sigma ,v},\;\;if\;\kappa_{1}^{\sigma ,v}=1,$$

(10) $${dlt}_{t}^{\sigma ,v}=\frac{{DS}_{t}^{\sigma ,v}\left(d{vr}_{t}^{\sigma ,v}\right)}{{dr}_{t}^{\sigma ,v}}\;\leq \;{dd}^{\sigma ,v},\;\;\;t_{0}^{\sigma ,v}\;\leq \;t\;<\;t_{e}^{\sigma ,v},\;\;if\;\kappa_{1}^{\sigma ,v}+\kappa_{2}^{\sigma ,v}=1,$$

(11) $$QL\left({ur}_{t}^{\sigma ,v}\right)=log\left(\frac{{ur}_{t}^{\sigma ,v}}{\underline{UR}\left(u{vr}_{t}^{\sigma ,v}\right)}\right),\;\;t_{0}^{\sigma ,v}\;\leq \;t\;<\;t_{e}^{\sigma ,v},\;\;if\;\kappa_{1}^{\sigma ,v}=1,\;\;$$

(12) $$QL\left({dr}_{t}^{\sigma ,v}\right)=log\left(\frac{{dr}_{t}^{\sigma ,v}}{\underline{DR}\left(d{vr}_{t}^{\sigma ,v}\right)}\right),\;\;\;\;\;\;t_{0}^{\sigma ,v}\;\leq \;t\;<\;t_{e}^{\sigma ,v},\;\;if\;\kappa_{1}^{\sigma ,v}+\kappa_{2}^{\sigma ,v}=1,$$

(13) $$QD\left({br}_{s}^{\sigma ,v}\right)=log\left(\frac{{dr}_{\tau_{s}}^{\sigma ,v}}{\underline{DB}\left({br}_{s}^{\sigma ,v}\right)}\right),\;{\;\;\;\;\;\;pt}_{1}^{\sigma ,v}\;\leq \;\tau_{s}\;<\;{pt}_{s}^{\sigma ,v},\;\;\;\;1\;<\;s\;\leq \;S^{\sigma ,v},\;\;\;if\;\kappa_{3}^{\sigma ,v}=1,$$

(14) $$u{vr}_{t}^{\sigma ,v}\geq {\underline{vr}}^{v},\;t_{0}^{\sigma ,v}\;\leq \;t\;<\;t_{e}^{\sigma ,v},\;\;if\;\kappa_{1}^{\sigma ,v}=1,$$

(15) $$d{vr}_{t}^{\sigma ,v}\geq {\underline{vr}}^{v},\;t_{0}^{\sigma ,v}\;\leq \;t\;<\;t_{e}^{\sigma ,v},\;\;if\;\kappa_{1}^{\sigma ,v}+\kappa_{2}^{\sigma ,v}=1,$$

(16) $${br}_{s}^{\sigma ,v}\geq {\underline{br}}^{v},\;1\;<\;s\;\leq \;S^{\sigma ,v},\;\;if\;\kappa_{3}^{\sigma ,v}=1,$$

(17) $${pt}_{s}^{\sigma ,v}\geq {pt}_{s-1}^{\sigma ,v}+\frac{{SS}_{s}^{\sigma ,v}\left({br}_{s}^{\sigma ,v}\right)}{{dr}_{\tau_{s}}^{\sigma ,v}},\;\;{pt}_{1}^{\sigma ,v}\;\leq \;\tau_{s}\;<\;{pt}_{s}^{\sigma ,v},\;\;\;\;1\;<\;s\;\leq \;S^{\sigma ,v},\;\;if\;\kappa_{3}^{\sigma ,v}=1,$$

(18) $${rbt}_{s}^{\sigma ,v}=\left\{\begin{array}{cc} \frac{{SS}_{s}^{\sigma ,v}\left({br}_{s}^{\sigma ,v}\right)}{{dr}_{\tau_{s}}^{\sigma ,v}}-{rbt}_{s-1}^{\sigma ,v}, & \frac{{SS}_{s}^{\sigma ,v}\left({br}_{s}^{\sigma ,v}\right)}{{dr}_{\tau_{s}}^{\sigma ,v}}\;>\;{rbt}_{s-1}^{\sigma ,v},\;\;if\;\kappa_{3}^{\sigma ,v}=1\\ 0 & otherwise \end{array}\right.,$$

(19) $${rbt}_{1}^{\sigma ,v}=0,\;\;\;\;if\;\kappa_{3}^{\sigma ,v}=1,$$

(20) $$\sum_{\vartheta }{ub}_{t}^{\vartheta ,\sigma }\geq {ur}_{t}^{\sigma ,v}\geq \underline{UR}\left(u{vr}_{t}^{\sigma ,v}\right),\;\;t_{0}^{\sigma ,v}\;\leq \;t\;<\;t_{e}^{\sigma ,v},\;\;if\;\kappa_{1}^{\sigma ,v}=1,$$

(21) $$\sum_{\vartheta }{db}_{t}^{\vartheta ,\sigma }\geq {dr}_{t}^{\sigma ,v}\geq \underline{DR}\left(d{vr}_{t}^{\sigma ,v}\right),\;\;t_{0}^{\sigma ,v}\;\leq \;t\;<\;t_{e}^{\sigma ,v},\;\;if\;\kappa_{1}^{\sigma ,v}+\kappa_{2}^{\sigma ,v}=1,$$

(22) $$\sum_{\vartheta }{db}_{\tau_{s}}^{\vartheta ,\sigma }\geq {dr}_{\tau_{s}}^{\sigma ,v}\geq \underline{DB}\left({br}_{s}^{\sigma ,v}\right),\;\;\;{pt}_{1}^{\sigma ,v}\;\leq \;\tau_{s}\;<\;{pt}_{s}^{\sigma ,v},\;\;\;1\;\leq \;s\;\leq \;\;S^{\sigma ,v},\;\;\;if\;\kappa_{3}^{\sigma ,v}=1,$$

(23) $${buf}_{\tau_{s}}^{\rho }+{SS}_{s}^{\sigma ,v}\left({br}_{s}^{\sigma ,v}\right)\;\leq \;{\bar{buf}}^{\rho },\;\;{pt}_{1}^{\sigma ,v}\;\leq \;\tau_{s}\;<\;{pt}_{s}^{\sigma ,v},\;\;\;\;1\;<\;s\;\leq \;S^{\sigma ,v},\;\;if\;\kappa_{3}^{\sigma ,v}=1.$$

Equation (8) allocates the required bandwidth for the video application in terms of the EV user’s perceived video quality. Equations (9) and (10) delineate the upload and download durations for video conferencing/live video during each time slot. Equations (11) and (12) present the distinct quality metrics utilized to assess the effectiveness of video conferencing and live video, following the guidelines outlined in the cited reference [61]. Furthermore, Equation (13) defines the quality metric applicable to VoD, as referenced in the related source [62].

Equations (14)–(16) ensure that the video resolution remains equal to or exceeds the minimum resolution defined by the video service provider. Simultaneously, Equation (17) guarantees the complete pre-download of individual video segments before initiating their playback. Moreover, Equations (18) and (19) delineate the rebuffering time for each video segment.

Equations (20) and (21) establish the prerequisite that the base stations covering the road segments traversed by the electric vehicle (EV) must possess the necessary upload and download bandwidth to support video conferencing and live video, as required. Similarly, Equation (22) imposes an analogous requirement for VOD. Additionally, Equation (23) ensures that the buffer space within the EV is appropriately dimensioned to accommodate a video segment before its playback.

To be specific, the optimization problem outlined in Equation (8) is being approximated in this scenario by lowering the requested video resolution from the EV user to align with the minimum resolution established by the video service provider.

Step 5: Should the bandwidth obtained in the previous step continue to be inadequate for supporting the minimum resolution specified by the video service provider, the EV user will receive a notification.

Step 6: This module operates in the background execution mode. Following the time interval established by the system, the process returns to Step 2 to readjust the quality and bandwidth prerequisites of the video application, provided that the video application remains active.

### 2.3. Real-Time Bandwidth Allocation for RSUs

If the video bandwidth requirement of the EV user cannot be met when the EV arrives at the road section managed by the RSU, this module assists in allocating the bandwidth according to the characteristics of the video application. If the user’s video application is video conferencing or live video, the bandwidth originally allocated for other VoD downloads in the same time slot will be reallocated to the required video conferencing or live video. Should the video bandwidth requirement remain unfulfilled, the EV will assume the role of the ultimate member within a fleet. Subsequently, the fleet will undergo expansion until the leading EV member enters a road segment where the base station’s coverage can sufficiently accommodate the demanded video bandwidth. The bandwidth is then transferred from the leading EV member to the requesting EV through V2V communication.

In the case of VoD, if a video segment cannot be downloaded in time before the video segment is played, the RSU checks the routes of other EVs that arrive at the same road section at the same time as the EV playing the VoD to find out whether there is a base station that can provide bandwidth for pre-downloading the required video segment. Once a base station that can support bandwidth is found, the EV traveling through the road section covered by the base station will download the required video segment to the on-board storage of the EV. When the EV storing the downloaded video segment arrives at the same road section as the requesting EV, the video segment can be transmitted to the VoD user via V2V communication.

The steps of this module are described below.

Step 1: For VoD, proceed to Step 8.

Step 2: The RSU allocates the bandwidth of other VoD(s) in the same time slot to the demanding video conferencing/live video.

Step 3: If the bandwidth requirement for video conferencing/live video is satisfied, go to Step 6. Otherwise, proceed to the next step.

Step 4: In cases where the bandwidth necessary for video conferencing or live video is inadequate, the RSU initiates the process by including the EV as the final member of a fleet for bandwidth transfer and incorporating other EVs into a fleet. This fleet expansion persists until the foremost fleet member reaches a road segment where the base station’s coverage can sufficiently supply the required bandwidth for the requesting EV. The establishment of the fleet must fulfill the subsequent requirement:

(24) $$\underset{P^{\sigma ,v}}{arg}Min\sum_{1\;\leq \;i\;<\;P^{\sigma ,v}}\left|(x_{r_{i}^{\sigma ,v}},y_{r_{i}^{\sigma ,v}})-\left(x_{r_{i+1}^{\sigma ,v}},y_{r_{i+1}^{\sigma ,v}}\right)\right|,\;if\;\kappa_{1}^{\sigma ,v}+\kappa_{2}^{\sigma ,v}=1,$$

subject to:

(25) $$R_{t}^{\sigma ,v}=\left[\left(r_{1}^{\sigma ,v},r_{2}^{\sigma ,v}\right),\cdots ,\left(r_{i-1}^{\sigma ,v},r_{i}^{\sigma ,v}\right),\cdots ,\left(r_{i-1}^{\sigma ,v},r_{P^{\sigma ,v}}^{\sigma ,v}\right)\right],\;\;t_{0}^{\sigma ,v}\;\leq \;t\;<\;t_{e}^{\sigma ,v},\;\mathrm{if}\;\kappa_{1}^{\sigma ,v}+\kappa_{2}^{\sigma ,v}=1,$$

(26) $$r_{P^{\sigma ,v}}^{\sigma ,v}=\sigma ,\;\mathrm{if}\;\kappa_{1}^{\sigma ,v}+\kappa_{2}^{\sigma ,v}=1,$$

(27) $${at}_{p_{1}^{\sigma }}\;\leq \;t_{0}^{\sigma ,v}\;\leq \;t_{e}^{\sigma ,v}\;\leq \;{at}_{p_{h^{\sigma }}^{\sigma }},\;\mathrm{if}\;\kappa_{1}^{\sigma ,v}+\kappa_{2}^{\sigma ,v}=1,$$

(28) $$\sum_{\vartheta }{ub}_{t}^{\vartheta ,r_{1}^{\sigma ,v}}\geq \underline{UR}\left({uvr}_{t}^{\sigma ,v}\right),\;\;t_{0}^{\sigma ,v}\;\leq \;t\;<\;t_{e}^{\sigma ,v},\;\;if\;\kappa_{1}^{\sigma ,v}=1\;\&\;\sum_{\vartheta }{ub}_{t}^{\vartheta ,\sigma }\;<\;\underline{UR}\left({uvr}_{t}^{\sigma ,v}\right),\;$$

(29) $$\sum_{\vartheta }{db}_{t}^{\vartheta ,r_{1}^{\sigma ,v}}\geq \underline{DR}\left({dvr}_{t}^{\sigma ,v}\right),\;\;t_{0}^{\sigma ,v}\;\leq \;t\;<\;t_{e}^{\sigma ,v},\;\;if\;\kappa_{1}^{\sigma ,v}+\kappa_{2}^{\sigma ,v}=1\;\&\;\sum_{\vartheta }{db}_{t}^{\vartheta ,\sigma }\;<\;\underline{DR}\left({dvr}_{t}^{\sigma ,v}\right),$$

(30) $${ut}_{t}^{r_{i+1}^{\sigma ,v},r_{i}^{\sigma ,v}}\geq \underline{UR}\left(u{vr}_{t}^{\sigma ,v}\right),\;t_{0}^{\sigma ,v}\;\leq \;t\;<\;t_{e}^{\sigma ,v},\;\;1\;\leq \;i\;<\;P^{\sigma ,v}\;\;if\;\kappa_{1}^{\sigma ,v}=1\;\&\;\sum_{\vartheta }{ub}_{t}^{\vartheta ,\sigma }\;<\;\underline{UR}\left({uvr}_{t}^{\sigma ,v}\right),$$

(31) $${dt}_{t}^{r_{i+1}^{\sigma ,v},r_{i}^{\sigma ,v}}\geq \underline{DR}\left(u{vr}_{t}^{\sigma ,v}\right),\;t_{0}^{\sigma ,v}\;\leq \;t\;<\;t_{e}^{\sigma ,v},\;1\;\leq \;i\;<\;P^{\sigma ,v}\;\;if\;\kappa_{1}^{\sigma ,v}+\kappa_{2}^{\sigma ,v}=1\;\&\;\sum_{\vartheta }{db}_{t}^{\vartheta ,\sigma }\;<\;\underline{DR}\left({dvr}_{t}^{\sigma ,v}\right).$$

Equation (24) serves as the foundation for configuring the fleet established during the transmission relay, optimized for the minimum fleet length. Building upon this, Equation (25) outlines the specific composition of the fleet, where Equation (26) identifies the designated EV as the terminal component of this fleet. To guarantee a harmonized sequence, Equation (27) specifies the scheduling of the initiation time for video conferencing or live video subsequent to the EV’s departure, while also ensuring the timely conclusion of the application before the EV reaches its destination.

Equations (28) and (29) guarantee that the leading fleet member reaches a road segment where the base station’s coverage can adequately provide the necessary bandwidth for the requesting EV. Additionally, Equations (30) and (31) establish that the V2V communication bandwidth between two successive fleet members must not fall below the relayed bandwidth acquired by the requesting EV.

To provide specific details, the process of forming the fleet as defined in Equation (24) entails a step-by-step assessment of all EVs that reach the same road segment as the requesting EV. Starting from the selected EV, the expansion of the fleet persists until the farthest member of the fleet enters a road segment where the coverage from the base station is proficient in supplying the required bandwidth for the EV that initiated the request.

Step 5: In instances where a certain VoD allocates bandwidth to video conferencing/live video, resulting in an inability to meet the minimum bandwidth requirement of the VoD due to reduced bandwidth availability, this module advances to the subsequent step in order to acquire the necessary bandwidth for the VoD. Conversely, the module concludes.

Step 6: To examine whether there are base stations in the coverage area of the road sections that can provide bandwidth for EVs to pre-download the required video segment(s):

(32) $$\sum_{\vartheta }{db}_{\tau_{s}}^{\vartheta ,\sigma }\geq \underline{DB}\left({br}_{s}^{\sigma ,v}\right),\;\;\;{pt}_{1}^{\sigma ,v}\;\leq \;\tau_{s}\;<\;{pt}_{s}^{\sigma ,v},\;\;1\;\leq \;s\;\leq \;\;S^{\sigma ,v},\;\;if\;\kappa_{3}^{\sigma ,v}=1,$$

(33) $${pt}_{s}^{\sigma ,v}\geq {pt}_{s-1}^{\sigma ,v}+\frac{{SS}_{s}^{\sigma ,v}\left({br}_{s}^{\sigma ,v}\right)}{\underline{DB}\left({br}_{s}^{\sigma ,v}\right)},\;\;\;\;\;\;1\;<\;s\;\leq \;\;S^{\sigma ,v},\;\;if\;\kappa_{3}^{\sigma ,v}=1,$$

(34) $${buf}_{\tau_{s}}^{\sigma }+{SS}_{s}^{\sigma ,v}\left({br}_{s}^{\sigma ,v}\right)\;\leq \;{\bar{buf}}^{\sigma },\;\;{pt}_{1}^{\sigma ,v}\;\leq \;\tau_{s}\;<\;{pt}_{s}^{\sigma ,v},\;\;\;\;1\;<\;s\;\leq \;\;S^{\sigma ,v},\;\;if\;\kappa_{3}^{\sigma ,v}=1.$$

Step 7: If bandwidth is available in the base station coverage area of another road section, pre-download the video segment from the base station to the on-board storage of the EV while passing through the base station coverage area. Notify the managing RSU of the base station and this module ends. If not, proceed to the next step.

Step 8: Check if other EVs arrive at the same road section as EV σ at time ${pt}_{s}^{\sigma ,v}$.

Step 9: If it is not possible to find an EV that arrives at the same time on the same road section as EV σ, the RSU notifies the EV user and this module ends. Instead, proceed to the next step.

Step 10: View the routes of the EVs arriving at the same section of the road at the same time as σ, and check if there is a base station that can provide bandwidth for the EV to pre-download VoD segment for σ:

(35) $$\sum_{\vartheta }{db}_{\tau_{s}\prime }^{\vartheta ,\rho }\geq \underline{DB}\left({br}_{s}^{\sigma ,v}\right),\;\;\;{pt}_{1}^{\sigma ,v}\;\leq \;\tau_{s}\prime \;<\;{pt}_{s}^{\sigma ,v},\;\;1\;\leq \;s\;\leq \;S^{\sigma ,v},\;\;if\;\kappa_{3}^{\sigma ,v}=1,$$

(36) $${pt}_{s}^{\sigma ,v}\geq {pt}_{s-1}^{\sigma ,v}+\frac{{SS}_{s}^{\sigma ,v}\left({br}_{s}^{\sigma ,v}\right)}{\underline{DB}\left({br}_{s}^{\sigma ,v}\right)},\;\;\;\;1\;<\;s\;\leq \;\;S^{\sigma ,v},\;\;if\;\kappa_{3}^{\sigma ,v}=1,$$

(37) $${buf}_{\tau_{s}\prime }^{\rho }+{SS}_{s}^{\sigma ,v}\left({br}_{s}^{\sigma ,v}\right)\;\leq \;{\bar{buf}}^{\rho },\;\;{pt}_{1}^{\sigma ,v}\;\leq \;\tau_{s}^{\prime }\;<\;{pt}_{s}^{\sigma ,v},\;\;\;1\;<\;s\;\leq \;S^{\sigma ,v},\;\;if\;\kappa_{3}^{\sigma ,v}=1,$$

(38) $${dt}_{\tau_{s}\prime }^{\sigma ,\rho }\geq \underline{DB}\left({br}_{s}^{\sigma ,v}\right),\;{pt}_{1}^{\sigma ,v}\;\leq \;\tau_{s}\prime \;<\;{pt}_{s}^{\sigma ,v},\;\;\;\;1\;<\;s\;\leq \;S^{\sigma ,v},\;\;if\;\kappa_{3}^{\sigma ,v}=1.$$

Step 11: If EV ρ that supports pre-downloading is found, the VoD segment will be pre-downloaded by ρ, and then will be transferred to the requesting EV via V2V communication when two EVs meet. If not, the RSU notifies the EV user and this module ends.

## 3. Experimental Results and Discussion

In this paper, the proposed algorithm is simulated using a personal computer with an Intel Core i7 2.9 GHz CPU and 64 GB RAM. To assess the effectiveness of the proposed algorithm, two comparison targets are employed. The first target is the conventional first-come, first-served (FCFS) method. The second reference benchmark is a contemporary state-of-the-art algorithm that combines the DQN and AQCS [2]. Remarkably, taking into account the prevailing trend in recent literature that commonly employs the strategy of pre-downloading video segments at base stations and RSUs to enhance video service quality for EV users, this paper specifically opts to evaluate the latest DQN+AQC algorithm as the second comparative target.

The historical traffic data are obtained from a website of traffic volume counts for New York City [63]. Video applications are divided into three categories, namely, video conferencing, live video, and VoD. Video conferencing and live video utilize AVC [6]. VoD uses VVC [10] with a bit rate half that of HEVC. Each video application has different bandwidth requirements; this paper refers to the bandwidths suggested by Skype [64], and Table 1 shows the download and upload bandwidths required for video conferencing. In addition, Table 2 shows the bandwidth required for live video by referring to the literature on the use of AVC [65,66]. For VoD, the required bandwidth is shown in Table 3 by referring to the literature on the use of HEVC [66] and considering half of the bandwidth required for video using HEVC in the literature as the bandwidth required for video using VVC.

Three types of base stations, including THz [28], mmWave [67], and sub-6 GHz [68], were set up in the simulated area to provide the bandwidth required for the video applications. Table 4 shows the bandwidth and transmission distance that can be provided by the three types of base stations. In addition, for V2V communication using VLC, this paper cites the relevant literature that involves simulations incorporating the use of car lights [45]. Additionally, Table 5 provides an overview of the variable bandwidths of VLC at different distances, distinguishing between daytime and nighttime circumstances.

**Figure 3:**
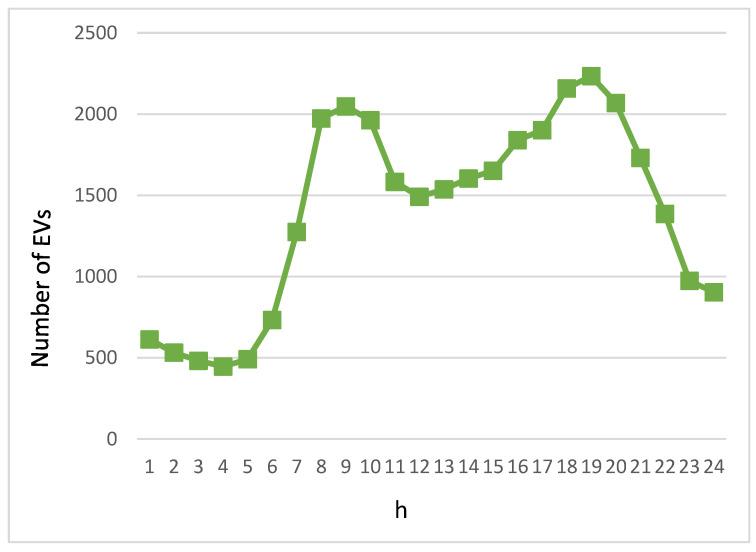
Volume of EVs within a day.

Figure 3 shows the number of EVs traveling on the road during a day in our simulation. It can be observed that there is a spike in traffic during the morning and evening peak hours. The number of EVs starts to increase from 06:00 until the morning peak is reached at 09:00. After the morning peak, the traffic volume stays above 1500 vehicles. The number of EVs starts to increase slightly after 16:00, and then the traffic volume starts to decrease after the evening peak is reached at 19:00, with the lowest number of EVs in the early morning hours.

**Figure 4:**
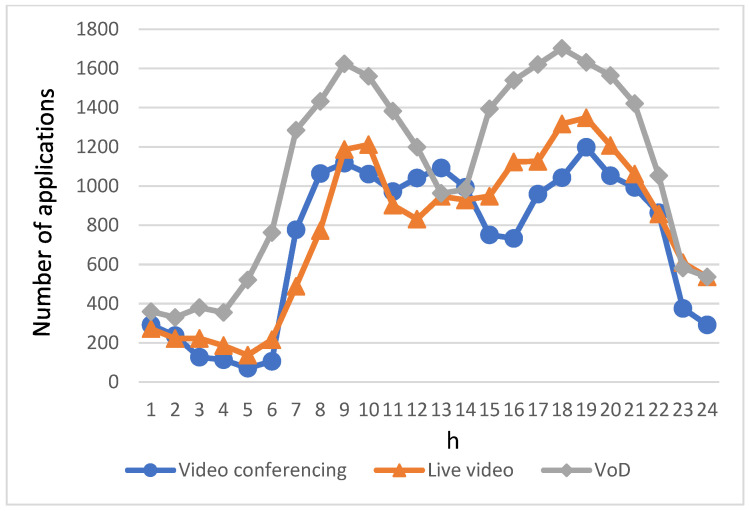
Number of users utilizing video conferencing, live video, and VoD within a day.

Figure 4 gives the user count of video applications within a day. As aforementioned, there are three types of video applications: video conferencing, live video, and VoD. It can be seen that the user counts of each application follow the same trend as the number of EVs. A small number of users still use video applications during the off-peak hours in the early hours of the morning, while the number of applications increases dramatically during the morning and evening peak traffic hours, when EV users have longer commute times during congestion and therefore have a greater demand for video applications. In addition, since VoD has the characteristics of flexible viewing time, repeatable viewing, and content diversity, it can be observed from Figure 4 that the number of users using VoD is higher than the number of users using video conferencing and live video in most of the time periods, no matter whether these are during peak or off-peak hours.

**Figure 5:**
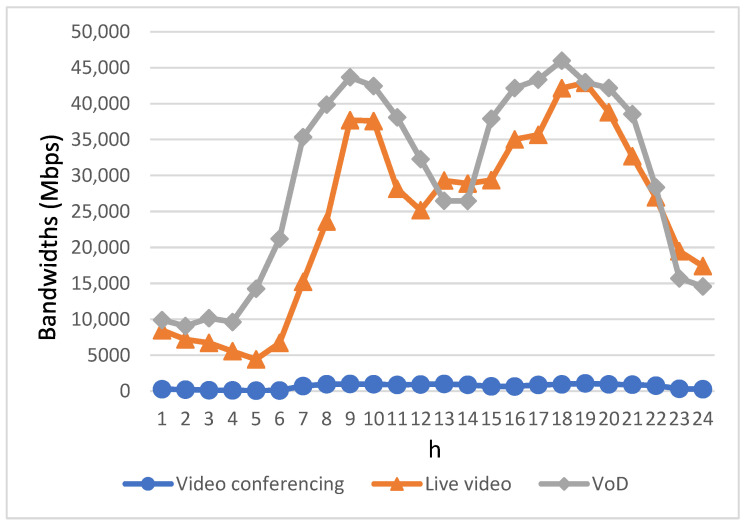
Bandwidth requirements for video conferencing, live video, and VoD within a day.

Figure 5 shows the video application bandwidth demand of users within a day. The bandwidth required for video conferencing is very low, as can be seen from Table 1. Even for high resolution, only 1.5 Mbps is required. Accordingly, even during the three peak hours of video conferencing users shown in Figure 4, the bandwidth required is still far less than the off-peak bandwidth demand of live video and VoD users. In addition, it can be seen from Figure 5 that the bandwidth demand for the use of live video and VoD is directly proportional to the number of their respective subscribers, with a significant increase in bandwidth demand during the morning and evening peak hours. Notably, although the number of users watching VoD is mostly higher than the number of users watching live video, as seen from Figure 4, the bandwidth requirement of VoD, as shown in Figure 5, is not much higher than that of live video, and even less than that of live video in several time slots.

**Figure 6:**
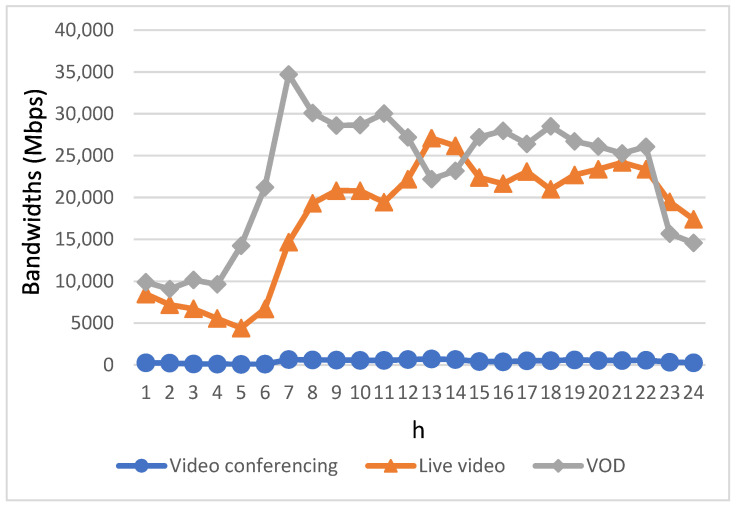
Bandwidth allocation for three types of video applications using the FCFS method.

Figure 6 shows the bandwidth obtained by the users of each video application using the conventional FCFS method. Observing the data, it becomes evident that during the early morning hours when users’ bandwidth requirements are minimal, the bandwidth accessible to EV users for each video application adheres to a direct proportionality with both their individual bandwidth demands and the sequence in which the users initiate their respective service applications. Due to the utilization of an FCFS strategy for bandwidth allocation by the base stations, applications initiated later in time struggled to secure sufficient bandwidth for real-time usage, particularly during peak periods. As a result, it is evident from the graph that, beginning at 08:00 and continuing until 22:00, the obtained bandwidth by EV users across different video applications does not align closely with the bandwidth requirements of these applications, as shown in Figure 5. Furthermore, the available bandwidth proves inadequate in fulfilling the requisite bandwidth for these applications, with the pronounced gap of insufficiency becoming notably conspicuous, particularly during the two peak periods of heightened user bandwidth demand.

**Figure 7:**
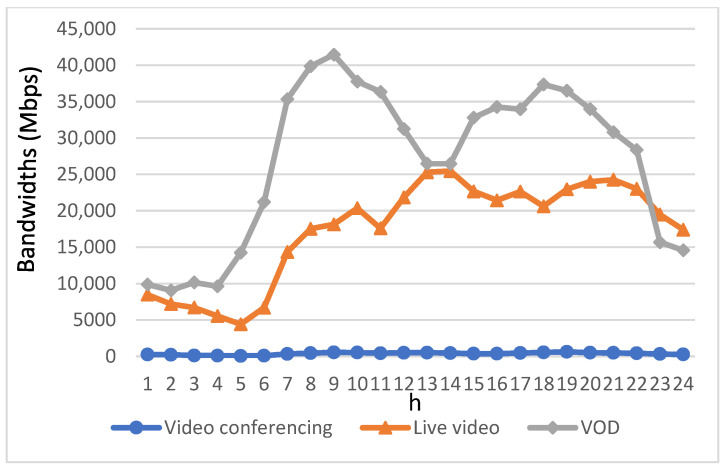
Bandwidth allocation for three types of video applications using the DQN+AQCS.

Figure 7 illustrates the bandwidth allocation for each video application using the DQN+AQCS algorithm. As seen in Figure 7, during the early morning and midnight hours when users have lower bandwidth requirements, the allocated bandwidth for EV users aligns with their individual demands, similar to the FCFS approach. However, the DQN+AQCS algorithm consistently enhances the available bandwidth for EV users across various time intervals when compared to the FCFS algorithm. Notably, starting at 08:00 and continuing until 22:00, the DQN+AQCS algorithm leverages the video pre-download mechanisms at RSUs and base stations, resulting in a significant increase in the available bandwidth for EV users compared to the FCFS method. This improvement is particularly pronounced for VoD applications, where video segments can be pre-downloaded at RSUs and base stations before their scheduled playback time. Nevertheless, it is important to acknowledge that the provided bandwidth still falls short of meeting the demands of EV users during the peak demand period characterized by heightened bandwidth requirements, even though urgent applications were prioritized. Consequently, both users of live video and VoD applications experienced insufficient bandwidth during rush hours. The performance of live video applications was particularly affected because there is no provision for pre-downloading in this type of application.

**Figure 8:**
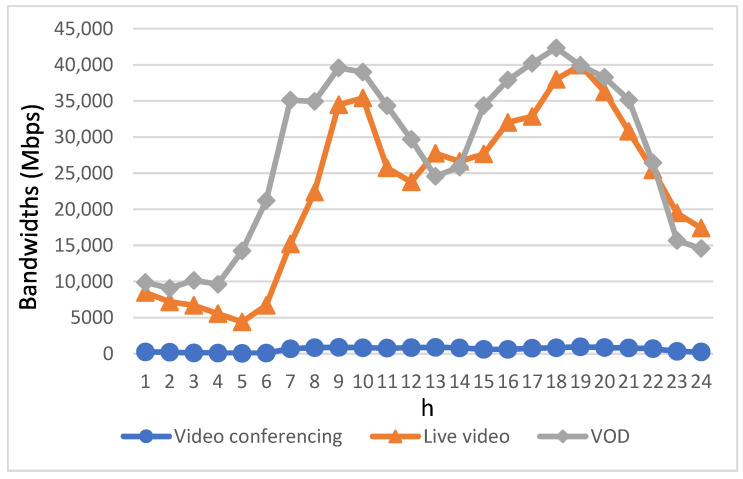
Bandwidth allocation for three types of video applications using the proposed algorithm.

Figure 8 illustrates the bandwidth assigned to each video application after using the proposed algorithm. As expected, the bandwidth available to each video service application is proportional to their demanded bandwidth, and the available bandwidth is significantly increased during the peak of bandwidth demand after applying the proposed algorithm. To provide further clarity, in cases where the bandwidth demand of a video conferencing or live video application cannot be satisfied, the “Real-time Bandwidth Allocation” mechanism implemented at the RSU was triggered into action. This activation facilitates the acquisition of bandwidth for the specific video application in need. Initially, the bandwidth allocated for other VoD downloads within the same time slot is repurposed to cater to the demanding video’s needs in a priority manner. In cases where the bandwidth requirement of the requesting video remains unfulfilled, there exists an alternative solution: bandwidth from base stations in other road sections can be reallocated to serve the requesting video’s needs. This process is facilitated through the cooperative efforts of EVs that assist in transmitting bandwidth via V2V communications. Concerning VoD applications, the proposed algorithm engages in the process of identifying a suitable EV capable of pre-downloading the required video segment onto its onboard storage prior to its encounter with the requesting EV. Subsequently, when these two EVs cross paths on the same road section, the pre-downloaded video segment is then efficiently transmitted to the requesting EV through the utilization of V2V communication mechanisms.

**Figure 9:**
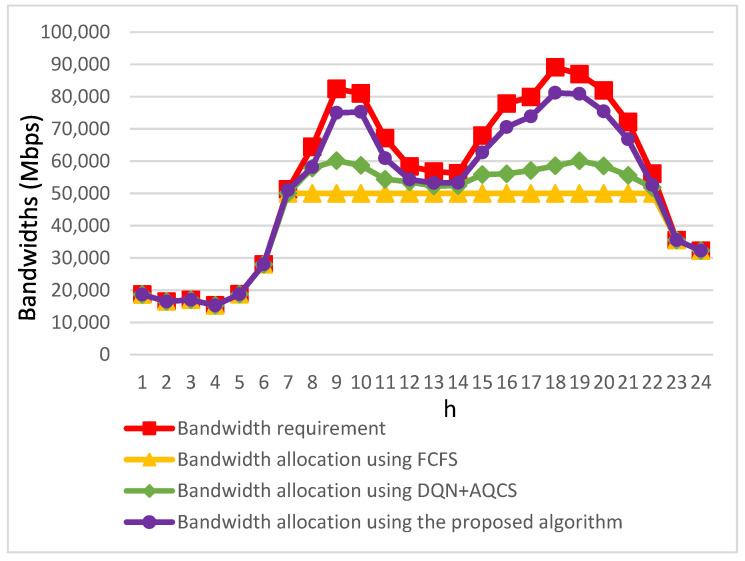
Comparative analysis of allocated bandwidth among the three approaches.

Figure 9 illustrates various curves, each conveying distinct aspects of bandwidth requirements and allocation for video applications. The curves are color-coded as follows:

- The red curve represents the bandwidth requirements of video applications.
- The yellow curve depicts the bandwidth allocated to video applications using the conventional FCFS method.
- The green curve showcases the bandwidth allocated to video applications when employing the DQN+AQCS algorithm.
- Finally, the purple curve demonstrates the bandwidth allocation achieved by video applications when utilizing the proposed algorithm.

The yellow curve in Figure 9 vividly illustrates that video applications employing the FCFS method exhaust the available bandwidth provided by base stations in congested road segments entirely between 08:00 and 22:00. Consequently, there is an insufficient amount of bandwidth remaining to accommodate the bandwidth requests of applications initiated later during this congested timeframe. This limitation stems from the fact that applications launched earlier within this time frame have already consumed a significant portion of the available bandwidth. Comparatively, as indicated by the green curve in Figure 9, the DQN+AQCS algorithm offers a substantial improvement in the bandwidth accessible to EV users between 08:00 and 22:00. This enhancement can be attributed to the algorithm’s implementation of a pre-downloading mechanism involving base stations and RSUs before the scheduled video segment playback.

As observed in the purple curve in Figure 9, when contrasting the proposed algorithm with the other two target algorithms, it consistently outperforms them during the high-bandwidth demand periods in the morning and evening. A significant portion of the required bandwidth for live video applications is efficiently delivered through real-time V2V communication. This process facilitates the seamless transfer of bandwidth from less congested road sections. The performance improvement of VoD applications involves the cooperative participation of other EVs in pre-downloading necessary video segments while passing by base stations with ample bandwidth. This adaptive approach proposed in the paper results in a considerable augmentation of the bandwidth allocated to each video application for EV users’ requests, particularly during the two peak time periods. In sum, the proposed algorithm not only effectively addresses the bandwidth demands of most video applications, but also significantly enhances the utilization of the bandwidth within base stations located in less-congested road sections.

**Figure 10:**
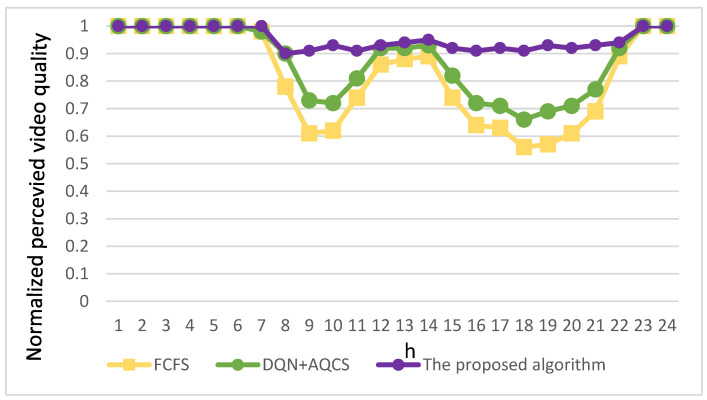
Comparative analysis of normalized perceived video quality among the three approaches.

Figure 10 provides a visual comparison, offering insights into the normalized perceived video quality among EV users and enabling a clear assessment of the relative effectiveness of these approaches. Notably, during the time frame from 23:00 to 07:00 of the subsequent morning, a substantial portion of users’ bandwidth demand is met. This occurs because EV users typically have reduced bandwidth requirements during late-night and early morning periods. Consequently, there is minimal impact on the perceived video quality for all three methods—FCFS, DQN+AQCS, and the proposed algorithm—during this time interval.

However, as depicted by the yellow curve in Figure 10, the FCFS method performs poorly due to a significant bandwidth deficit during high-bandwidth-demand periods. Although the utilization of the DQN+AQCS algorithm, represented by the green curve in Figure 10, helps alleviate the reduction in perceived video quality for EV users during high-bandwidth-demand periods, it encounters a challenge of unstable perceived video quality between 08:00 and 22:00, especially during two peak time periods. Specifically, for the two compared approaches, the average bandwidth available to EV users decreases proportionally with the rise in video bandwidth demand within congested road sections, where the base station’s bandwidth allocation has reached its upper limit. A significant drop in the quality of video usage by EV users is evident during peak hours. Only during the midday hours, when the video bandwidth scarcity is less severe, does the perceived video quality for EV users improve.

Conversely, as shown on the purple curve in Figure 10, the proposed algorithm effectively addresses these challenges by utilizing V2V communication to relay bandwidth from base stations with surplus capacity to the requesting live video applications and by efficiently pre-downloading video segments as requested by VoD applications. To offer a more detailed perspective, Figure 9 reveals that the proposed algorithm leads to only a slight decline in user-perceived video quality between 08:00 and 22:00. When compared to the FCFS method during the same time frame, it delivers a substantial average improvement of 30% in perceived video quality for EV users. Moreover, during the morning and evening peak hours, particularly at 09:00 and 19:00, this enhancement becomes even more significant, with improvements reaching 49% and 63%, respectively. In contrast to the DQN+AQCS approach, there is an average improvement of 17%. The most significant enhancements are observed at 10:00 and 18:00, where improvements reach 29% and 37%, respectively.

## 4. Conclusions

Although some studies in the literature have proposed bandwidth allocation algorithms to provide video services to EV users, there are no bandwidth allocation algorithms for video services that utilize emerging communication technologies for next-generation vehicular networks. In view of this, this paper integrates the THz base stations of 6G wireless networks with the existing mmWave and sub-6 GHz band base stations to provide the required bandwidth for EV users. A bandwidth management mechanism that suits the video quality requirements of different EV users is proposed to allocate the bandwidth from the three types of base stations mentioned above to the in-vehicle video services. Meanwhile, VLC technology is utilized in V2V communications to provide a high data rate and high security for inter-vehicle video transmissions.

A series of simulations is conducted to comprehensively compare the performance of the proposed algorithm against both the conventional FCFS method and a contemporary state-of-the-art approach known as DQN+AQCS. This rigorous evaluation process allowed for a thorough assessment of how the proposed algorithm performed in comparison to these two benchmark methods. The simulation results clearly demonstrated the efficacy of the proposed mechanisms, including video pre-downloading for EV users and bandwidth relay, in conjunction with the utilization of VLC for V2V communication. These mechanisms collectively enhanced the adaptability of base station bandwidth allocation, particularly during peak hours. This enhancement ensures that video conferencing, live video, and VoD applications for EV users can reliably access the necessary bandwidth support even in scenarios of bandwidth scarcity on congested road segments during peak times. Ultimately, the perceived video quality for EV users can be maintained at satisfactory levels.

In summary, the algorithm presented in this paper offers a dual advantage. Specifically, it effectively addresses the challenge of bandwidth contention among EV users, which stems from the constraints of base station bandwidth. Additionally, it represents a notable advancement over both the FCFS and DQN+AQCS approaches. To be more precise, it ensures access to sufficient bandwidth for real-time live video applications and guarantees uninterrupted playback of the vast majority of video applications even during periods of heightened demand. This enhancement significantly elevates the overall quality of the video viewing experience for EV users. Remarkably, even when the available bandwidth from congested base stations reaches its peak capacity, the proposed algorithm demonstrates a substantial improvement of 30% and 17% in average video quality compared to the FCFS approach and the DQN+AQCS method, respectively. This further solidifies the effectiveness of our proposed algorithm in providing satisfactory video quality even within the constraints of bandwidth limitations.

## Figures and Tables

**Table 1:** Parameters of required bandwidth for video configuration with AVC.

Video Format	Download Required Bandwidth	Upload Required Bandwidth
Screen Sharing	300 Kbps	300 Kbps
High Quality	500 Kbps	500 Kbps
High Definition	1.5 Mbps	1.5 Mbps

**Table 2:** Parameters of required bandwidth for live video with AVC.

Video Format	Download Required Bandwidth
720 p 30 fps	3 Mbps
1080 p 60 fps	17 Mbps
2160 p 60 fps	60 Mbps

**Table 3:** Parameters of required bandwidth for VoD with VVC.

Video Format	Download Required Bandwidth
1080 p 60 fps	7.5 Mbps
2160 p 60 fps	20 Mbps
4320 p 60 fps	50 Mbps

**Table 4:** Parameters of bandwidth available for VLC in different situations.

Distance	Daytime Bandwidth	Nighttime Bandwidth
10 m	2790 Mbps	2810 Mbps
100 m	336 Mbps	362 Mbps

**Table 5:** Parameters of bandwidth available for THz, mmWave, and sub-6 GHz base stations.

Type of Base Station	Bandwidth	Transmission Distance
THz	54~24 Gbps	39 m
MmWave	2~1.8 Gbps	150 m
Sub-6 GHz	1~0.5 Gbps	622 m

## Data Availability

Not applicable.

## Nomenclature

$p_{1}^{\sigma }$	Starting point of the driving route of EV σ
$p_{i}^{\sigma }$	The *i*th road section of the traveling route of EV σ
$p_{h^{\sigma }}^{\sigma }$	The final destination of the EV σ
${at}_{p_{i}^{\sigma }}$	The time when EV σ reaches road section $p_{i}^{\sigma }$
SVR(·)	The predicted travel time of EV from $p_{i}^{\sigma }$ to $p_{i+1}^{\sigma }$ using SVR
${sp}_{p_{i}^{\sigma },p_{i+1}^{\sigma }}\left({at}_{p_{i}^{\sigma }}\right)$	The average speed at the time of ${at}_{p_{i}^{\sigma }}$ arriving at the road section connecting $p_{i}^{\sigma }$ and $p_{i+1}^{\sigma }$
$\rho_{p_{i}^{\sigma },p_{i+1}^{\sigma }}\left({at}_{p_{i}^{\sigma }}\right)$	The traffic volume passing through the road segment connecting $p_{i}^{\sigma }$ and $p_{i+1}^{\sigma }$ at the time of ${at}_{p_{i}^{\sigma }}$
${wd}_{p_{i}^{\rho },p_{i+1}^{\rho }}\left({at}_{p_{i}^{\sigma }}\right)$	The day of the week on which the segment connecting $p_{i}^{\sigma }$ and $p_{i+1}^{\sigma }$ is traversed
${wt}_{p_{i}^{\rho },p_{i+1}^{\rho }}\left({at}_{p_{i}^{\sigma }}\right)$	The weather condition at the time of ${at}_{p_{i}^{\sigma }}$
$\kappa_{1}^{\sigma ,v}$	Whether the video application is a video conferencing application
$\kappa_{2}^{\sigma ,v}$	Whether the video application is a live video application
$\kappa_{3}^{\sigma ,v}$	Whether the video application is a VoD application
${ub}_{t}^{\vartheta ,\sigma }$	The upload bandwidth that base station ϑ allocates for EV σ at time slot *t*
${db}_{t}^{\vartheta ,\sigma }$	The download bandwidth that base station ϑ allocates for EV σ at time slot *t*
$\underline{UR}\left({uvr}_{t}^{\sigma ,v}\right)$	The minimum bandwidth requirement for uploading of video application v at time *t*
$\underline{DR}\left({dvr}_{t}^{\sigma ,v}\right)$	The minimum bandwidth requirement for downloading of video application v at time *t*
${uvr}_{t}^{\sigma ,v}$	The video resolution chosen by the EV user for uploading
${dvr}_{t}^{\sigma ,v}$	The video resolution chosen by the EV user for downloading
${\underline{vr}}^{v}$	The minimum resolution for video conferencing or live video set by the software application provider
${\underline{br}}^{v}$	The minimum resolution for VoD v set by the software application provider
$S^{\sigma ,v}$	The number of selected video segment for VoD v
$\tau_{s}$	The download time of selected video segment *s*
${pt}_{s}^{\sigma ,v}$	The playback time of selected video segment *s*
${SS}_{s}^{\sigma ,v}\left({br}_{s}^{\sigma ,v}\right)$	The length of VoD segment *s* when the bit rate is ${br}_{s}^{\sigma ,v}$
${db}_{\tau_{s}}^{\vartheta ,\sigma }$	The bandwidth that base station ϑ allocates for the VoD segment in the time slot $\tau_{s}$
$t_{0}^{\sigma ,v}$	The time when the video conferencing/live video application starts
$t_{e}^{\sigma ,v}$	The time when the video conferencing/live video application ends
${pt}_{1}^{\sigma ,v}$	The start time of the VoD application
${pt}_{S^{\sigma ,v}}^{\sigma ,v}$	The end time of the VoD application
${buf}_{\tau_{s}}^{\sigma }$	The remaining buffer of σ at $\tau_{s}$
${\bar{buf}}^{\sigma }$	The buffer size of σ
${ult}_{t}^{\sigma ,v}$	The upload times of video conferencing/live video at time slot *t*
${dlt}_{t}^{\sigma ,v}$	The download times of video conferencing/live video at time slot *t*
${ud}^{\sigma ,v}$	The maximum upload delay limit for video conferencing/live video at time *t*
${dd}^{\sigma ,v}$	The maximum download delay limit for video conferencing/live video at time *t*
$\phi^{\sigma ,v}$	The weight of the lower bound of delay for video transmission in Equation (8)
$\xi^{\sigma ,v}$	The weight of the video inter-frame transmission smoothness penalty in Equation (8)
${rbt}_{s}^{\sigma ,v}$	The rebuffering of the selected video segment of the video application
$\psi^{\sigma ,v}$	The weight of rebuffering in Equation (8)
${sd}^{\sigma ,v}$	The starting delay time of the video application
$\omega^{\sigma ,v}$	The weight of starting delay time of the video application in Equation (8)
$\beta^{\sigma ,v}$	The weight of the inter-segment smoothness penalty of VoD v
${DS}_{t}^{\sigma ,v}\left(u{vr}_{t}^{\sigma ,v}\right)$	The video frame size for video conferencing/live video with uploading resolution ${uvr}_{t}^{\sigma ,v}$
${DS}_{t}^{\sigma ,v}\left(d{vr}_{t}^{\sigma ,v}\right)$	The video frame size for video conferencing/live video with downloading resolution ${dvr}_{t}^{\sigma ,v}$
${ur}_{t}^{\sigma ,v}$	The upload bit rate of v at time *t*
${dr}_{t}^{\sigma ,v}$	The download bit rate of v at time *t*
$\underline{DB}\left({br}_{s}^{\sigma ,v}\right)$	The minimum bandwidth requirement of the video segment *s* of VoD v when the preset bit rate is ${br}_{s}^{\sigma ,v}$
${dr}_{\tau_{s}}^{\sigma ,v}$	The actual bandwidth of VoD v downloaded at time $\tau_{s}$
${buf}_{\tau_{s}}^{\rho }$	The remaining buffer of EV ρ that downloads the segment for σ at $\tau_{s}$
${\bar{buf}}^{\rho }$	The buffer size of EV ρ
$R_{t}^{\sigma ,v}$	The fleet formed at time *t*
$P^{\sigma ,v}$	The number of EVs in the fleet
$x_{r_{i}^{\sigma ,v}}$	The x coordinate of the *i*th EV in the fleet
$y_{r_{i}^{\sigma ,v}}$	The y coordinate of the *i*th EV in the fleet
${ut}_{t}^{r_{i+1}^{\sigma ,v},r_{i}^{\sigma ,v}}$	The bandwidth uploaded from $r_{i+1}^{\sigma ,v}$ to $r_{i}^{\sigma ,v}$ at time *t*
${dt}_{t}^{r_{i+1}^{\sigma ,v},r_{i}^{\sigma ,v}}$	The bandwidth downloaded from $r_{i+1}^{\sigma ,v}$ to $r_{i}^{\sigma ,v}$ at time *t*
ρ	The EV that pre-downloads the VoD segment
${dt}_{\tau_{s}\prime }^{\sigma ,\rho }$	The bandwidth that EV σ downloads from EV ρ at time slot $\tau_{s}\prime$
